# A ″On–Off″ Fluorescent Sensor Based on Coumarin-Furoic Hydrazide for Recognition of Fe^3+^: Drinking Water, Test Strip Applications and DFT Calculations

**DOI:** 10.1007/s10895-025-04212-2

**Published:** 2025-02-26

**Authors:** Abdurrahman Karagoz, Tahir Savran, Ibrahim Yilmaz

**Affiliations:** 1 Department of Chemistry, Kamil Ozdag Science Faculty, Karamanoglu Mehmetbey University, 70100 Karaman, Türkiye; 2 Department of Chemistry and Chemical Processing Technologies, Vocational School of Technical Sciences, Karamanoglu Mehmetbey University, 70100 Karaman, Türkiye; 3 Department of Mathematics and Science Education, Faculty of Education, Bolu Abant Izzet Baysal University, 14030 Bolu, Türkiye; 4 Innovative Food Technologies Development Application and Research Centre, Bolu Abant Izzet Baysal University, 14030 Bolu, Türkiye

**Keywords:** Fluorescence sensor, Iron, Coumarin, Natural Water, DFT

## Abstract

A coumarin based fluorescent probe (E)-N'-((7-hydroxy-2-oxo-2H-chromon-3-yl)methylene)furan-2-carbohydrazide (**CFHZ**) was synthesized for the detection of Fe^3+^ and its characterizations were carried out using spectroscopic methods such as FT-IR, mass spectrometry^1^H-NMR, ^13^C-NMR. The novel probe **CFHZ** showed a highly selective and sensitive “turn-off” response to Fe^3+^ ion without any interference from other analytes. Strong fuorescence quenching phenomena of the **CFHZ** were observed in EtOH:H_2_O (99/1, v/v) detection system (λ_em_ = 470 nm) upon the additions of Fe^3+^. The binding stoichiometry between **CFHZ** and Fe^3+^ was determined by Job's method, FT-IR and MALDI TOF–MS and found to be 2:1. Also, the binding constant was determined to be 1.82 × 10^5^ M^−1^ and the limits of detection for the analysis of Fe^3+^ was measured as 25.7 nM. Besides, experimental applications were carried out for real-time monitoring of Fe^3+^ in water samples using developed sensor. Additionally, fluorescence imaging experiments for Fe^3+^ detection of **CFHZ** probe on test papers were successfully performed.

**Supplementary Information:**

The online version contains supplementary material available at 10.1007/s10895-025-04212-2.

## Introduction

A serious heavy metal pollution occurs in environmental waters today in consequence of the rapid development of industrialization [1, 2]. In addition, since heavy metals can accumulate intensely in fish or shellfish and drinking water, it is inevitable that these products will harm human health by disturbing the balance of the human body when these products are consumed as food [1, 2]. Therefore, the recent focus of biochemical and environmental research is the development of fluorescent sensors for the detection of environmentally and biologically important metal ions [3–10].

Along with copper, which is among the heavy metals, iron is considered a crucial trace metal for organisms and plays a vital role in many physiological and environmental systems [11–13]. However, the natural accumulation of iron in the food chain and its nonebiodegradation pose a serious threat to human health and the environment [11]. Iron is recognized to be the second most ubiquitous trace element in the human body. In addition, this element is a necessary ion for almost all living organisms and takes on important tasks in many basic metabolic and physiological processes such as DNA and RNA synthesis, proton and electron transfer, cofactor function in enzyme synthesis, osmotic regulation, neural transmission, transport and storage of oxygen and carbon dioxide, and protein storage [9, 12–21]. As a consequence of Fe^3+^ deficiency and excess iron intake in the body, a number of serious diseases such as anemia, Alzheimer's and Parkinson's disease, hemochromatosis, dysfunctions of various organs, huntington's disease, heart diseases, βethalassemia, diabetes, Friedreich's ataxia, hepatitis, cancer and even death may arise associated with oxidative stress [5, 6, 9–17, 19, 21–29]. Furthermore, it has been set by theWHO (World Health Organization) that the ultimate permissible level of iron in water is at 0.3 mg/L (roundly 6 μM) [15, 21].

The development of techniques for the determination of iron a heavy metal ion in real samples has always been regarded as a significant study because of its negative environmental and health effects [1, 5, 11]. To monitor trace Fe^3+^, various analytical techniques including atomic absorption/emission spectroscopy (AAS/AES), inductively coupled plasma mass/atomic emission spectrometry (ICP-MS/ICP-AES), gas chromatography, plasmon resonance Rayleigh scattering (PRRS) and magnetic resonance imaging (MRI) have hitherto been extensively utilised [1, 5, 10, 11, 13, 22]. Nevertheless, these classical methods have limited applicability in analyses because of high cost, unportable, wastage of electricity, long term analyses, specialized staff requirement and complicated tools [1, 12, 13, 15]. The development of cheap, rapid and easy-to-use detection techniques/instruments with high sensitivity/selectivity for the analysis of iron (III) has been widely studied and is still in demand [1].

In the scientific researches of the fluorescent chemosensors that can detect Fe^3+^, various mechanisms such as metal to ligand charge transfer (MLCT), photoinduced electron transfer (PET), photoinduced proton transfer (PPT), internal charge transfer (ICT), chelation-enhanced fluorescence (CHEF) effect, excited state intramolecular proton transfer (ESIPT) and C = N isomerization have been reported [6]. In this context, promising colorimetric and fluorescent detection processes have gained much attention with extensive applications in biological and environmental analysis because of their unique unparalleled advantages like excellent sensitivity and selectivity in aqueous and noneaqueous media, quick response time, costless equipage, on the spot monitoring, and ease of handling [1, 3, 6, 10–13, 15, 16, 30, 31]. Considering to these advantages, many studies focusing on the development of novel colorimetric and fluorescent sensors to identify Fe^3+^ have been investigated and published [11, 12, 14, 15, 17, 32]. Within this framework, a large number of chromogenic and fluorogenic chemical sensors based on a series of fluorophores such as BODIPY [33], indole [9], rhodamine [14, 17, 34], phosphazene [3, 8, 21], antracene [13], pyrenev [20], carbazole [11, 32] and coumarin [18, 35, 36] have been improved for determination of Fe^3+^ over the past decade. However, they have own disadvantages such as poor water-solubility, low selectivity or sensitivity, long response time and small stokes shift for the recognizing of Fe^3+^. So, the design and synthesis of a simple and effective colorimetric and fluorescent sensors for Fe^3+^ with high selectivity and good reversibility is rare and still a great need [5, 9, 11, 15, 18, 20, 32, 37–40]. As a fluorophore or chromophore, the coumarin ring has intense fluorescence behavior with high photostability, high quantum yield and large Stokes shift [41]. In recent years, studies of coumarin-based sensors that respond only to Fe^3+^ are rare [42–44].

In this context, in the present paper, a novel coumarin-based fluorescent “off” type chemosensor was designed and synthesized, which remarkably exhibited selective/sensitive detection of Fe^3+^ with a blue to colorless color change response over other metal ions in EtOH conditions. The chemosensor **CFHZ** was elucidated using ^1^H-NMR, ^13^C-NMR, FT-IR and mass spectrometry methods. The Fe^3+^ binding ratios and sensing mechanism of the chemosensor **CFHZ** were confirmed through FTIR spectra and Mass spectra. The results obtained from experimental studies of **CFHZ** and the Fe^3+^ complex were supported by theoretical calculations using density functional theory (DFT).

## Materials and Methods

### General Experimental Chemicals and Instruments

All chemicals and solvents under this study were obtained from Sigma-Aldrich (Sigma-Aldrich Co., USA), VWR Chemicals BDH Inc. (Pennsylvania, USA) and Merck (Germany) and were used without any purification. Stock solutions of metal ions (Hg^2+^; Fe^2+^; Fe^3+^; Cu^2+^; Zn^2+^; Cd^2+^; Al^3+^; K^+^; Co^2+^; Ni^2+^; Ag^+^; Pb^2+^; Ca^2+^; Mg^2+^; Ba^2+^) were prepared as 1.0 × 10^2−^ M) using perchlorate salts.

The ^1^H and ^13^C NMR, mass and infrared (ATR, 4000–650 cm^−1^) spectra of **CFHZ** were obtained using a 300 MHz NMR spectrometer (Bruker–DPX Massachusetts, CA, USA), mass spectrometer (Microflex LT MALDI TOF–MS) and Spectrum-100 infrared spectrophotometer (Perkin Elmer Inc., Waltham, Massachusetts, USA), respectively. In addition, emission and absorption spectra were recorded on Agilent Cary 60 UV–Vis and Agilent Cary Eclipse fluorescence device (Agilent Technologies, Inc, California, USA). The all emission photographs were taken under a portable cabin equipped with UV–lamps (λ_ex_ = 365 nm) using an Apple iPhone 6S model phone (Apple Inc., California, USA). The pH measurements were carried out using a bench top pH meter (Mettler Toledo, Zaventem, The Netherlands). A Milli-Qs®7003/05/10/15 water purification system (Merck KGaA) was used for ultrapure water.

### Syntheses

**Scheme 1:**
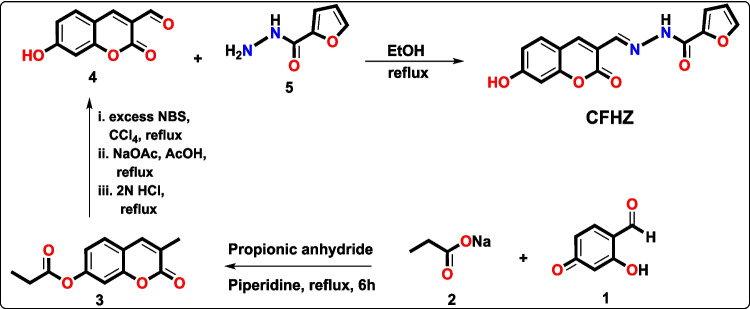
The synthetical procedure of the chemosensor **CFHZ**

As seen in Scheme 1, compounds **3** and **4** were synthesised according to the literature [45] and detailed procedures were included in the supporting information.

To obtain **CFHZ,** to a solution of 7-Hydroxy-2-oxo-2H-chromene-3-carbaldehyde **4** (100 mg, 0.525 mmol) dissolved in 25 mL of ethanol was added ethanolic solution of furan-2-carbohydrazide **5** (79.59 mg, 0.631 mmol). The mixture was refluxed overnight and after cooling to room temperature a yellow solid was obtained. After filtration, the solid was washed with cold ethanol successively and dried in vacuo to obtain (E)-N'-((7-hydroxy-2-oxo-2H-chromon-3-yl)methylene)furan-2-carbohydrazide (**CFHZ**) with 91.5% yield (1.89 g).

^**1**^**H NMR (300 MHz, DMSO-d**_**6**_**)** δ (ppm) 11.99 (s, 1H), 10.84 (s, 1H), 8.49 (s, 1H), 8.44 (s, 1H), 7.94 (s, 1H), 7.76 (dt, *J* = 8.6, 1.2 Hz, 1H), 7.31 (s, 1H), 6.83 (dt, *J* = 8.7, 2.0 Hz, 1H), 6.75 (d, *J* = 2.0 Hz, 1H), 6.69 (dt, *J* = 3.6, 1.8 Hz, 1H). ^**13**^**C NMR (75 MHz, DMSO- d**_**6**_**)** *δ* (ppm) 163.10, 162.99, 161.00, 154.76, 150.73, 147.16, 146.71, 142.42, 131.92, 129.43, 117.07, 114.58, 112.86, 112.26, 102.76. Mass (*m*/*z*): found 298.049 and calculated 298.06 for compound **CFHZ**.

### Fluorescence Studies of the CFHZ

The spectral studies of the **CFHZ** compound were carried out by fluorescence spectroscopy in ethanol/water (99:1, v/v) media at 25 °C. For measurements, stock solutions of **CFHZ** and metals (Hg^2+^; Fe^2+^; Fe^3+^; Cu^2+^; Zn^2+^; Cd^2+^; Al^3+^; K^+^; Co^2+^; Ni^2+^; Ag^+^; Pb^2+^; Ca^2+^; Mg^2+^; Ba^2+^), were prepared at 1 × 10^–2^ M. Then, the concentration of the **CFHZ** in EtOH/H_2_O (99:1, v/v) was diluted to 5 × 10^–6^ M. Emission data were recorded in the region of 400–800 nm (λ_ex_ = 388 nm, λ_em_ = 470 nm, slit width 10/20 nm). To test the selectivity of **CFHZ**, the Fe^3+^ solution was transferred to **CFHZ** solution containing competitive metal ions and the emission intensities were measured. Furthermore, fluorescence titration studies of **CFHZ** were carried out by adding increasing amounts (0–16 eq.) of Fe^3+^ solution to the **CFHZ** solution. The binding stoichiometry of **CFHZ**-Fe^3+^ complex was studied by Job's plot method based on titration experiments. Besides, from the titration experiments, the Benesi–Hildebrand constant of **CFHZ** with Fe^3+^, LOD and LOQ values were calculated (LOD = 3xσ / K; LOQ = 10xσ / K) [46].

### Computational Study of CFHZ

DFT computational studies were carried out using the Gaussian 09W program (Wallingford CT, UK). For the optimization of **CFHZ** and **CFHZ**-Fe^3+^, the DFT method, Becke's three-parameter Lee–Yang–Par (B3LYP) function and the 6–31G (d,p) basis set were used [47–49]. Time Dependent Density Functional Theory (TD-DFT) was applied using optimized geometries in the gas phase to provide only positive eigenvalues were obtained.

### Applications of CFHZ

In order to observe the analytical performance of the developed sensor, two different applications were carried out on real samples. For this purpose, Fe^3+^ determination in drinking water was carried out using **CFHZ** probe and test paper application was also carried out.

The test papers were prepared by dropping **CFHZ** (5 × 10^–6^ M) solution in EtOH:H_2_O (99:1, v/v) onto TLC test strips and then dried in air. Then, the Fe^3+^ solution was dropped onto the papers and photographs were taken under UV lamp. In the application of Fe^3+^ determination in drinking water, 3 mL of **CFHZ** solution was taken into a quartz cuvette and the fluorescence intensity was measured. Later, 15 μL of drinking water filtered in a membrane filter (0.45 µm) was added to the **CFHZ** solution and the fluorescence intensity was measured. Then, this mixture was spiked with two different concentrations of Fe^3+^ solutions and fluorescence measurements were performed. Using the obtained results, the relative standard deviation and % recovery values were calculated.

## Results and Discussion

### Synthesis and Characterization of CFHZ

The synthesis of the chemosensor **CFHZ** was successfully performed as shown in Scheme 1. Compounds **3** and **4** in the scheme were synthesized according to the literature [45] and the structures of the synthesised compounds were clarified by ^1^H—^13^C NMR, FT-IR and MALDI-TOF MS spectra (Figs. S1-S5). The synthesis of compound 4 from compound 3 was carried out according to the methyl to aldehyde conversion reaction known as the Riley oxidation reaction [50]**.** In the synthesis phase of the chemosensor **CFHZ** (E)-N'-((7-hydroxy-2-oxo-2H-chromen-3-yl)methylene)furan-2-carbohydrazide, a Schiff base derivative, was designed and furnished with a 91.5% yield by refluxing the compound **4** and 2-furoic hydrazide **(5)** in EtOH. For the structural characterization of the chemosensor **CFHZ**, aromatic proton peaks in the coumarin moiety and furan ring resonated at 6.80 – 7.80 ppm in the ^1^H − NMR spectra (Fig S1). The singlet peaks appear at 11.99 and 10.84 ppm is attributed to the–NH and phenolic –OH group present respectively. In addition, proton signal of the imine (CH = N) was observed at 7.94 ppm. The carbonyl carbons (–C = O) of cyclic ester in the coumarin moiety and furoic hydrazide component were monitored at *δ* 163.10 and 161.00 ppm in ^13^C–NMR spectra (Fig S2). Besides, the mass (MALDI TOF–MS) spectral data showed that the structure was nearly matched to its exact molecular weight (Fig. S3).

### Fluorescence Studies of CFHZ

First of all, the solvent mixture in which we could obtain maximum emission intensity for **CFHZ** and maximum quenching for **CFHZ**—Fe^3+^ complex was determined. For this purpose, the emission intensities of **CFHZ** and **CFHZ**—Fe^3+^ complex solutions were measured in different solvents such as ethyl alcohol (EtOH), dimethyl sulfoxide (DMSO), dimethyl formamide (DMF), water (H_2_O), acetone (Ace) and acetonitrile (MeCN). In addition, the same experiments were carried out in organic solvent–water mixtures and the obtained results were given in Figure S6a,b. When Figures S6a and S6b were examined, it was seen that the most suitable solvent was EtOH and EtOH—H_2_O mixture ratio was (99:1, v/v) and therefore the fluorescence studies were carried out in [EtOH:H_2_O (99:1, v/v)].

**Fig. 1:**
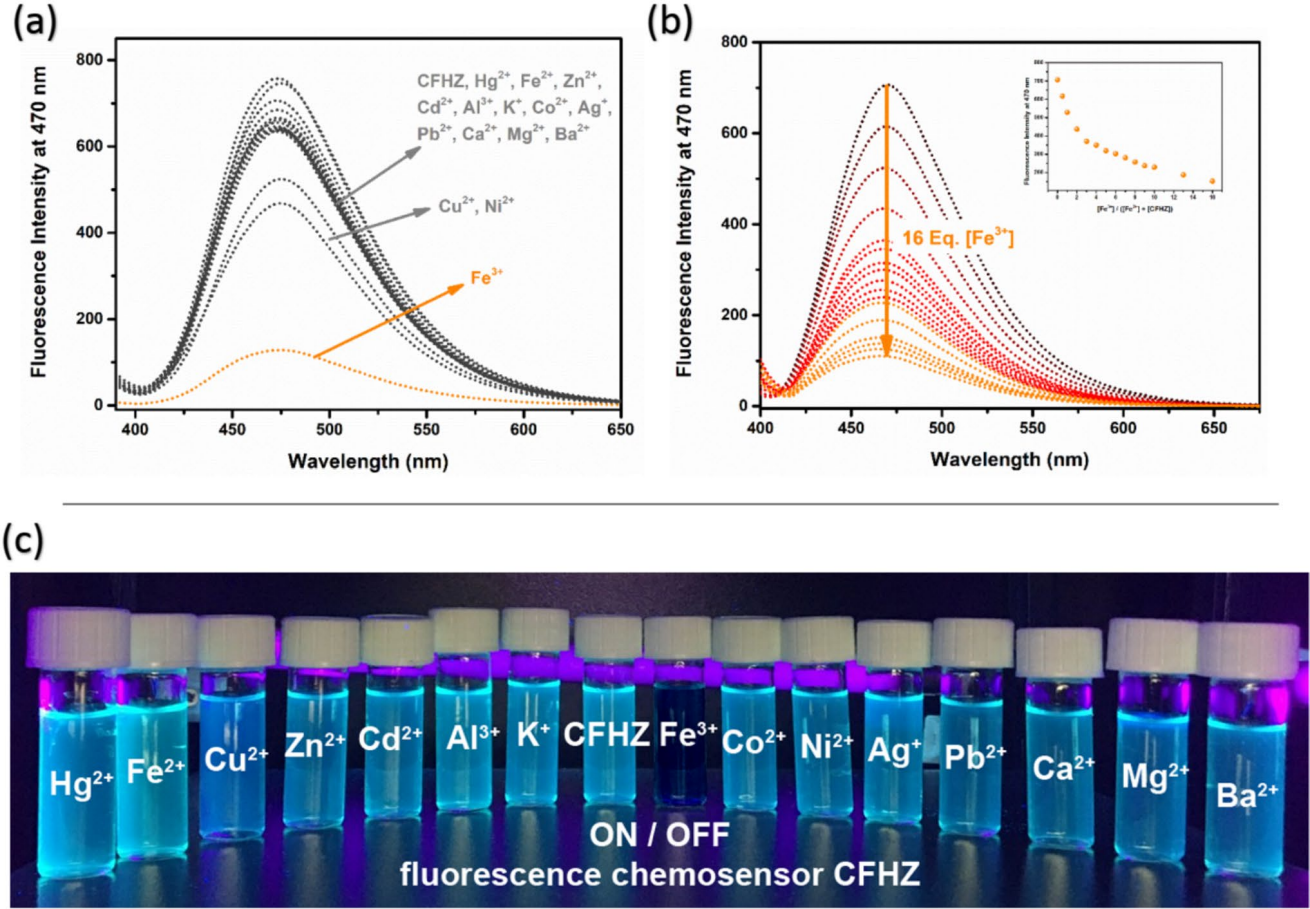
(**a**) Fluorescence response of probe **CFHZ** at 470 nm in the presence of various analytes in EtOH:H_2_O (99:1, v:v), (**b**) Fluorescence titration spectra of probe **CFHZ** upon successive addition of Fe^3+^ (0 – 16 eq.) in EtOH: H_2_O (99:1, v:v), and (**c**) the photograph of probe **CFHZ** taken under UV–lamp in the presence of various analytes

The fluorescence behaviour of **CFHZ** in the presence of cations was investigated in EtOH:H_2_O (99:1, v/v) medium at room temperature. While the addition of Fe^3+^ to the **CFHZ** allow of a significant decrease in the emission intensity of the sensor at 470 nm, the addition of Hg^2+^, Fe^2+^, Cu^2+^, Zn^2+^, Cd^2+^, Al^3+^, K^+^, Co^2+^, Ni^2+^, Ag^+^, Pb^2+^, Ca^2+^, Mg^2+^ and Ba^2+^ did not cause a notable decrease in the fluorescence intensity of the probe. Meanwhile, Cu^2+^ caused a small reduction in fluorescence intensity, which was negligible compared to Fe^3+^. Furthermore, the high response of **CFHZ** to Fe^3+^ was also observed under UV lamp at 365 nm (Fig. 1c). When a series of metal ions were added to **CFHZ** solution, it can be seen with the naked eye that only Fe^3+^ ion caused a color change from “light blue” to “colorless” under 365 nm UV lamp (Fig. 1c) indicating that our probe is operating as a “turn off” fluorescent probe for Fe^3+^. Then, for quantitative determination of Fe^3+^ with our probe, an emission titration study was performed by adding different concentrations (0—125 µM) of Fe^3+^ solution to **CFHZ** (Fig. 1b). The emission intensity of the probe at 470 nm gradually decreased with the increase of [Fe^3+^], and the minimum level was reached at 16 equivalents.

**Fig. 2:**
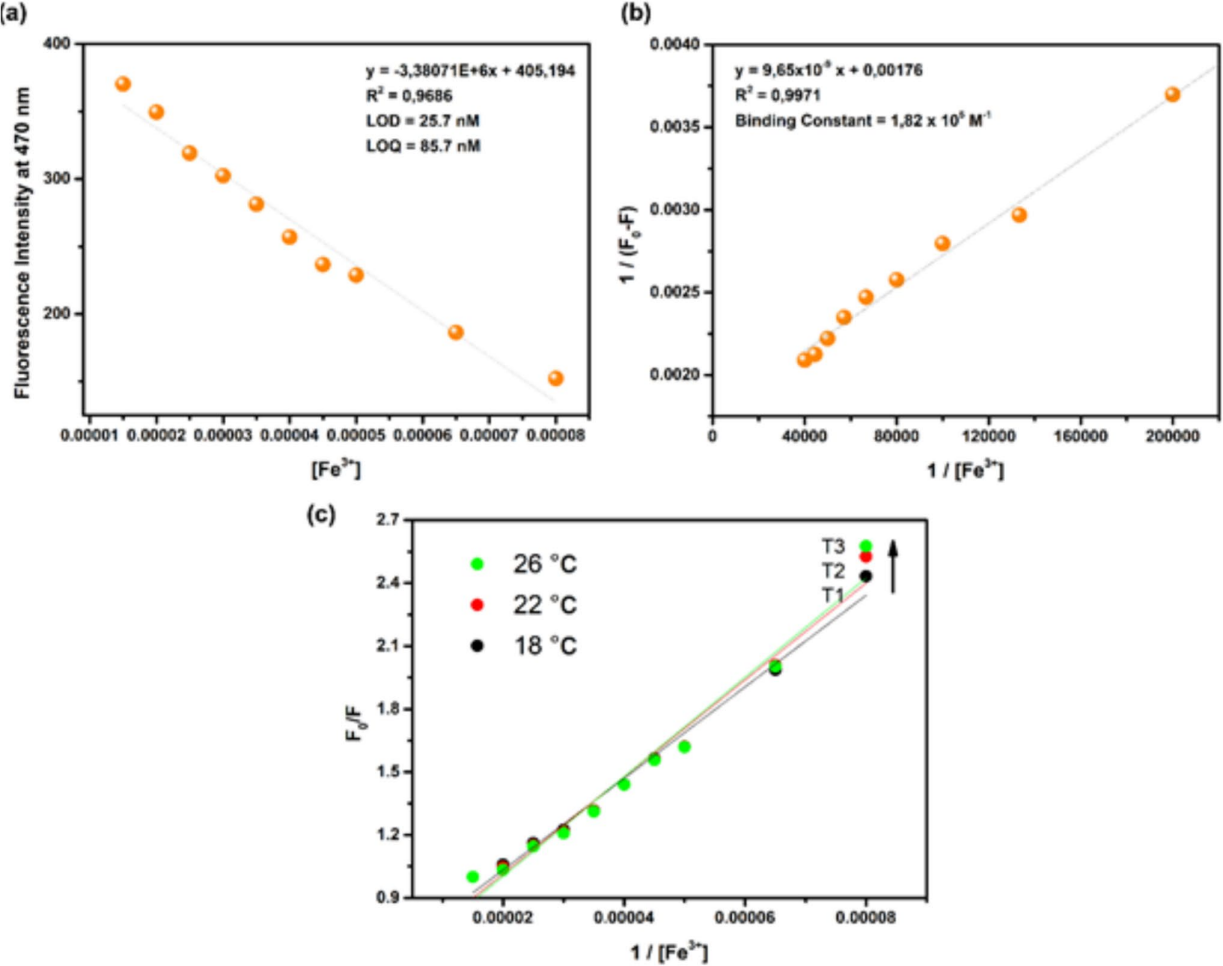
(**a**) Calibration plot for the fluorescence intensity of **CFHZ** (5 × 10^−6^ M) vs [Fe^3+^] and (**b**) Benesi–Hildebrand plot for the relative emission intensity [1 / (F_0_ – F)] of **CFHZ** (5 × 10^−6^ M) vs 1 / [Fe^3+^] [λ_ex_ = 388 nm; λ_em_ = 470 nm; EtOH: H_2_O (99:1, v:v) media)] (**c**) Fluorescence intensity ratio F_0_/F versus inverse concentration of Fe.^3+^ at working temperatures (T1 = 16 °C, T2 = 22 °C and T3 = 26 °C)

Binding constant is an important concept to understand and calculate when studying ligand–metal complexes. Therefore, using the fluorimetric titration data, the Benesi-Hildebrand plot of [1/(F_0_-F)] versus 1/[Fe^3+^] was plotted and the binding constant of Fe^3+^ to **CFHZ** was calculated. The Fig. 2b showed a good linearity with the high correlation coefficient (R^2^ = 0.9971) and binding constant was found as 1.82 × 10^5^ M^−1^. LOD and LOQ values were calculated from the calibration curve using the formulas LOD = 3xσ / k and LOQ = 10xσ / k (where σ is the standard deviation of the blank measurement and k is the slope of the calibration curve), and found to be 25.7 nM and 85.7 nM, respectively (Fig. 2a). The obtained LOD values are much lower than the maximum permissible level recommended by the World Health Organization (WHO) guidelines (5 μM) for Fe^3+^ in drinking water [15, 21]. The detection performance of our sensor with a low limit of detection (LOD) of Fe^3+^ was seen to be better compared to probes reported in the literature (Table 1).

**Table 1:**
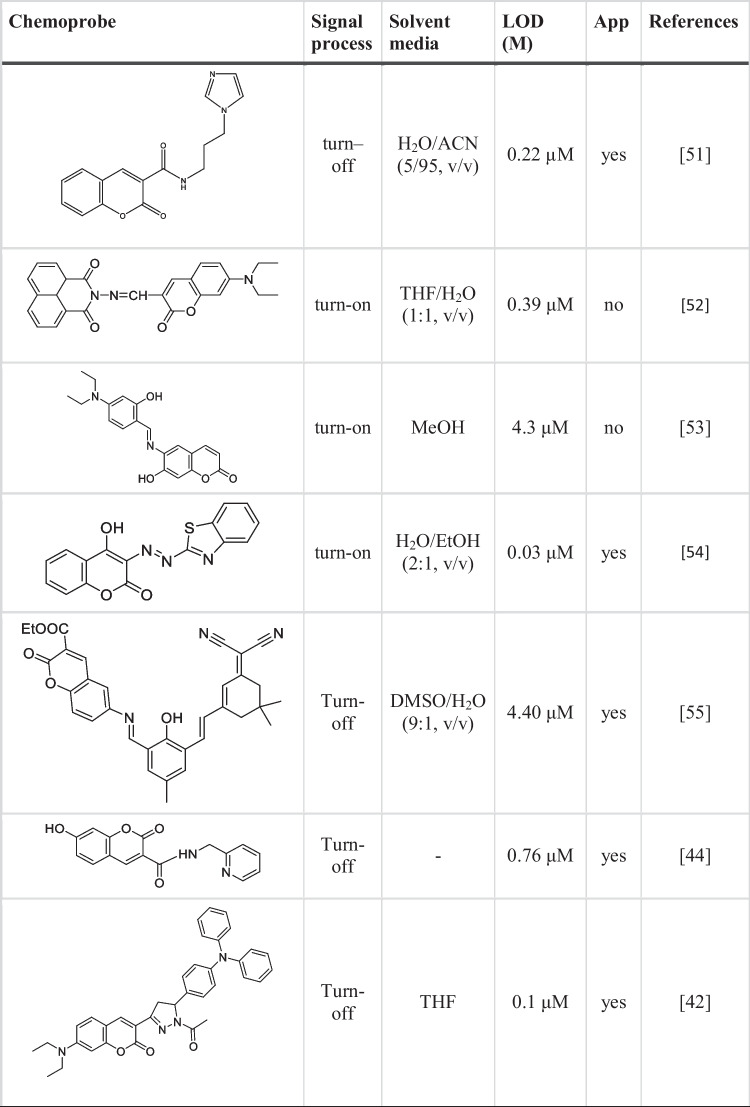

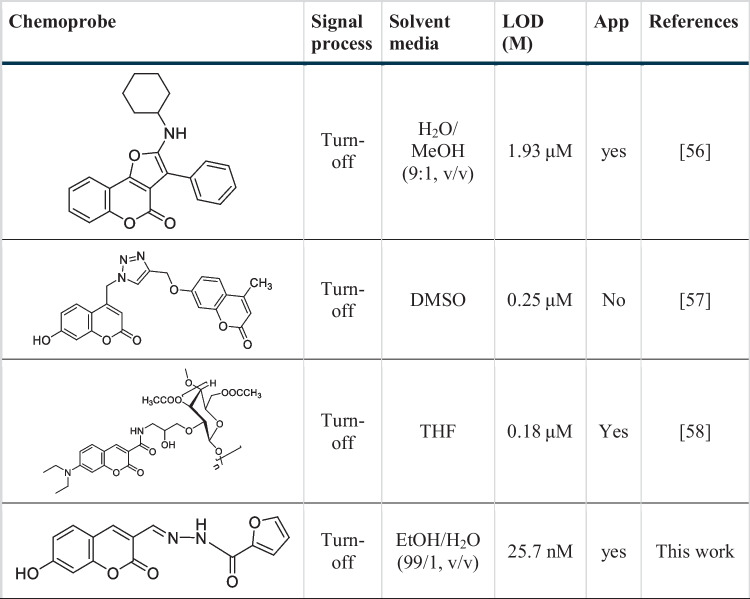
Comparison of the sensor performance of probe **CFHZ** with other Fe^3+^ sensors

**Fig. 3:**
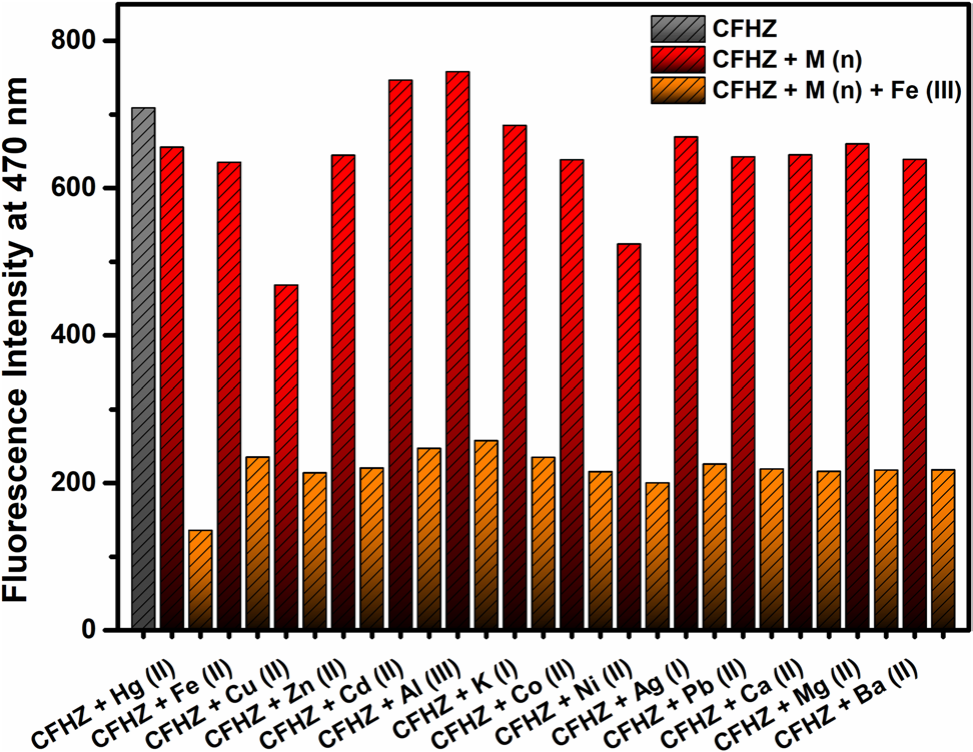
Fluorescent response of competing ions and competing ions + Fe^3+^ in probe **CFHZ** solution system

Selectivity is another feature of the sensor, which refers to its ability to distinguish between the target substance and other substances that may be present in the environment. Hence, the fluorescence intensities of **CFHZ** were measured by adding Fe^3+^ to the solution containing interfering ions (Hg^2+^; Fe^2+^; Cu^2+^; Zn^2+^; Cd^2+^; Al^3+^; K^+^; Co^2+^; Ni^2+^; Ag^+^; Pb^2+^; Ca^2+^; Mg^2+^; Ba^2+^). As seen in Fig. 3, there was no appreciable change in the fluorescence intensity of **CFHZ** in the presence of additional interfering ions (10.0 eq.) (red column), while a significant decrease was observed when Fe^3+^ (10.0 eq.) was added to the solutions of interfering cations (orange column). The results of these competition experiments demonstrate a high selectivity of **CFHZ** towards Fe^3+^ ions.

To determine the pH working range in which **CFHZ** can detect Fe^3+^ the emission intensities of **CFHZ**—Fe^3+^ solutions at different adjusted pHs (4–10) were recorded. As seen in Figure S7, no remarkable change was observed in the fluorescence intensity of **CFHZ** containing Fe^3+^ except at pH 4. These results revealed that the **CFHZ** probe can recognize Fe^3+^ ions in a broad pH range. To determine the response time of the developed probe, the fluorescence response was measured after adding Fe^3+^ to **CFHZ** and was found to be around ~ 30 s (Figure S8). In addition, the reversibility study for **CFHZ** was carried out with EDTA (10^–2^ M) (Figure S9).

### Stern–Volmer Calculations

The Stern–Volmer constant (K_sv_) is an indicator of metal ion binding efficiency and can be calculated using the following equation:

$${F}_{0}/F={k}_{SY}\left[Q\right]\,+\,1$$

where F_0_ and F represent the emission intensities of the **CFHZ** before and after the addition of Fe^3+^. K_sv_ is the Stern–Volmer constant and [Q] is the total Fe^3+^ concentration. Figure 2c shows the signal intensity ratio F_0_/F of **CFHZ** as a function of invers concentration Fe^3+^ at various temperatures (T1 = 16 °C, T2 = 22 °C and T3 = 26 °C). Stern–Volmer constants at three different temperatures were calculated as 2.18 × 10^4^ M^−1^, 2.31 × 10^4^ M^−1^ and 2.37 × 10^4^ M^−1^, respectively. The Stern–Volmer graphs were linear whose slopes increase slightly with temperature. Higher temperature result in the dissociation of weakly bound complexes, and hence smaller amounts of static quenching. The results indicated that the mechanism conforms dynamic quenching in **CFHZ**—Fe^3+^ complex [59].

### Binding Phenomenon (Mechanism) of CFHZ Against Fe^3+^

**Fig. 4:**
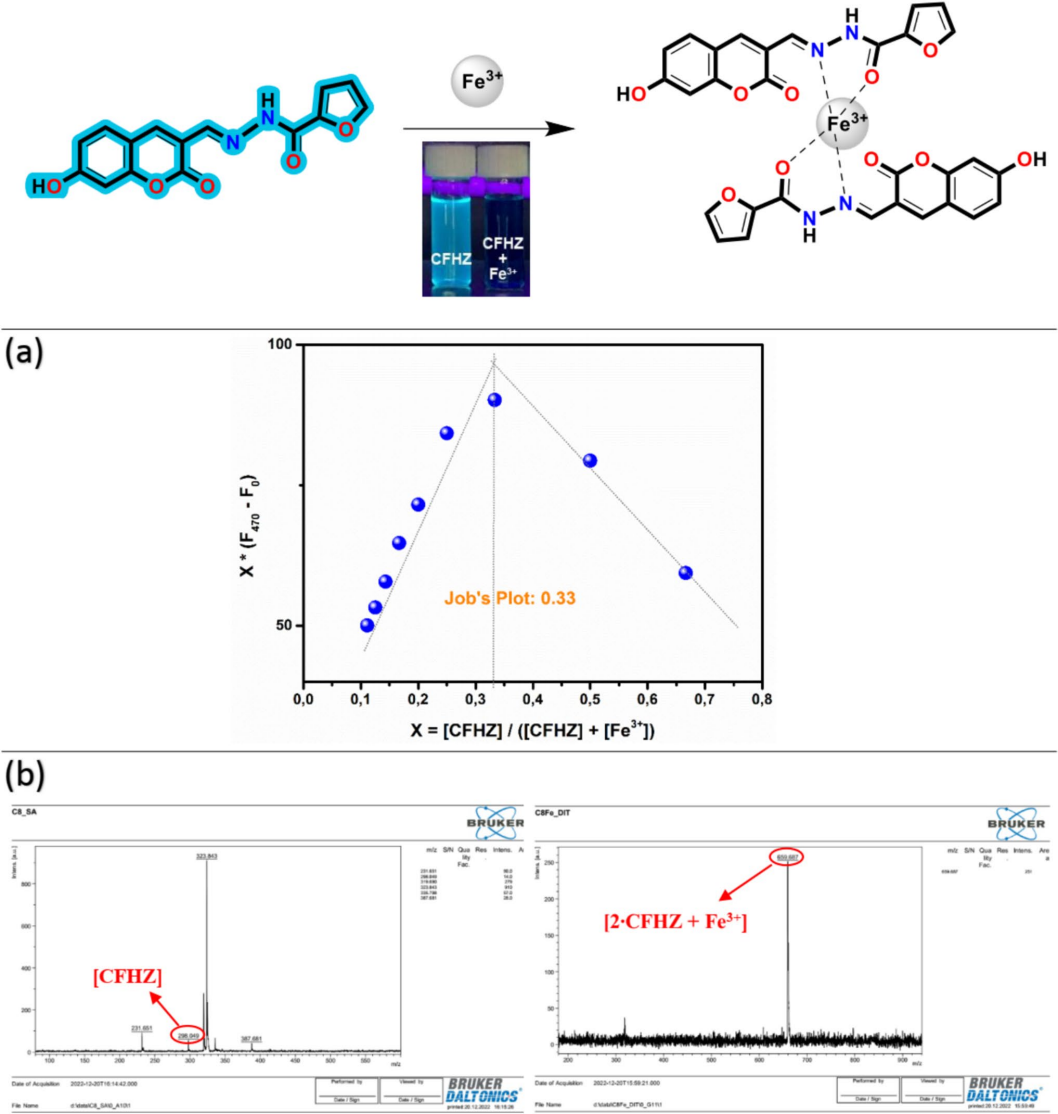
(**a**) Job’s graph for **CFHZ**-Fe^3+^ complex in EtOH:H_2_O (99:1, v:v) and (**b**) MALDI-TOF–MS spectrum of **CFHZ**-Fe^3+^ complex

MALDI TOF–MS, FT–IR and Job's plot methods were used to estimate the binding stoichiometry of the complexes between **CFHZ** and Fe^3+^. In the Job graph given in Fig. 4a, it is seen that the maximum emission for **CFHZ** – Fe^3+^ is at the mole fraction of 0.33, which corresponds to a ratio of 2:1 (**CFHZ** – Fe^3+^). The MALDI TOF–MS results given in Fig. 4b also support this situation. Looking at Fig. 4b, it is seen that the mass peaks at m/z 298.05 and 652.69 belong to the [**CFHZ**] and [2 × **CFHZ** + Fe^3+^] structures, respectively. In addition, FT-IR spectroscopy was used to elucidate the structure of the **CFHZ** – Fe^3+^ complex. When the FT-IR spectrum is examined, it is seen that there are shifts in the wavenumbers of the peaks at 1687 cm^−1^ and 1614 cm^−1^ (belonging to C = O and CH = N, respectively), which proves that the complexation occurs via the carbonyl and imine nitrogen. In conclusion, the above-mentioned results confirm that the proposed binding mechanism of **CFHZ** with Fe^3+^ occurs as shown in Fig. 4.

### Density Functional Theory (DFT) Calculations and Sensing Mechanism

DFT and TD-DFT calculations were carried out to support both the geometric, electronic structures of **CFHZ** and **CFHZ**—Fe^3+^ complexes determined by spectroscopic methods and to estimate the sensing mechanism. As seen in Fig. 1b, the emission intensity of the probe **CFHZ** gradually decreased with the addition of [Fe^3+^] ion and quenching occurred. The quenching of fluorescence intensity is due to the formation of **CFHZ**—Fe^3+^ complex, which can be attributed to the paramagnetic quenching effect known as the ligand to metal charge transfer mechanism (LMCT) [60].

**Fig. 5:**
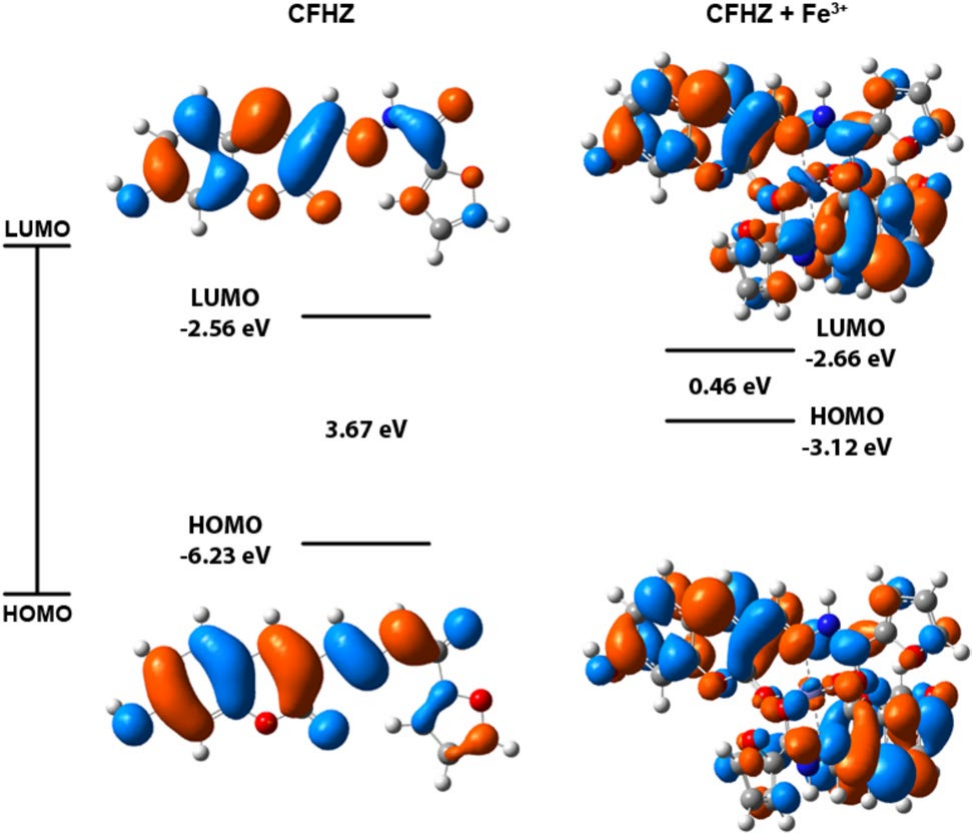
HOMO / LUMO orbitals of the **CFHZ** and **CFHZ** – Fe^3+^

The Fe^3+^ ion, with an unfilled d shell and paramagnetic nature, effectively quenches fluorescence through electron transfer processes. The transfer of electrons from the excited states of probe **CFHZ** to the d-orbitals of Fe^3+^ ions reduces light emission, leading to a significant decrease in fluorescence. In Fig. 5, it is seen that the electron density in **CFHZ** molecule is spread along the coumarin ring, while it is distributed throughout the **CFHZ**—Fe^3+^ molecule after the addition of Fe^3+^. According to TD-DFT calculations, the highest contribution to the electronic transitions for **CFHZ** is between HOMO—LUMO. HOMO orbitals are primarily located in the ligand π-system, while the LUMO orbitals are located in the metal center. As presented in Fig. 5, the energy gap between the HOMO and LUMO of **CFHZ** was 3.67 eV, while the energy gap of the **CFHZ**—Fe^3+^ complex decreased to 0.46 eV, indicating that the system became more stable with the addition of Fe^3+^. The decrease in the HOMO—LUMO energy gap supports the LMCT process.

### Implementations of CFHZ

After the characteristic parameters of the developed probe were determined by fluorescence studies, real-time tracking of Fe^3+^ in water samples and test papers was investigated in order to test its usability. The experiments were performed at least triplicates and the results were given in Table 2. The standard addition method was used for the determination of Fe^3+^ in drinking water samples and 0.10 and 0.20 mol.L^–1^ of Fe^3+^ solutions were added to the samples in the measurements. After adding Fe^3+^ to the **CFHZ** solution, fluorescence intensities were measured. The recovery value for added Fe^3+^ was found to be between 99.26—100.12% and the RSD values were found to be between 1.16—3.52%. These results showed that our probe **CFHZ** is a promising tool for the identification of Fe^3+^ in water samples. In addition, the probe was made into a test strip using TLC papers impregnated with **CFHZ** solution (Fig. S10a). "**CFHZ**" and "**CFHZ** + Fe(III)" were written on TLC papers with **CFHZ** solution and their photographs taken under a UV lamp (365 nm). When exposed to a solution of Fe^3+^, the **CFHZ** coated paper immediately showed a considerable colour change from blue to colourless under a 365 nm UV lamp (Fig. S10b). The application with TLC papers shows that **CFHZ** and Fe^3+^ can be determined qualitatively in a practical way.

**Table 2:** Recognition of Fe^3+^ in water samples by **CFHZ**

	spiked Fe^3+^ (μM)	found Fe^3+^ (μM)	recovery (%)	RSD (%)
Ultra pure water	0.0	0.00017 ± 0.000005		2.87
	0.5	0.500784 ± 0.010516	100.12	2.10
	1.0	1.000819 ± 0.019616	100.06	1.96
Natural spring water-1	0.0	0.002625 ± 0.000092		3.52
	0.5	0.498910 ± 0.005787	99.26	1.16
	1.0	1.001130 ± 0.024728	99.85	2.47
Natural spring water-2	0.0	0.002070 ± 0.000026		1.26
	0.5	0.500446 ± 0.012111	99.68	2.42
	1.0	1.001198 ± 0.025831	99.91	2.58

## Conclusion

In conclusion, a novel coumarin-based fluorescence probe was developed for Fe^3+^ detection in EtOH:H_2_O (99:1, v/v). The developed probe **CFHZ** behaved as a turn-off probe with high selectivity in the presence of competing ions and showed excellent detection performance for Fe^3+^ with significantly low detection limit (25.7 nM). Also, the binding stoichiometric ratio between **CFHZ** and Fe^3+^ was determined by Job's method, FT-IR and MALDI TOF–MS and found to be 2:1. The Fe^3+^ detection of the chemosensor was highly reversible and speedy, it was performed in less than 1 min. Furthermore, the chemosensor **CFHZ** exhibited perfect potential for selective and sensitive sensing for Fe^3+^ in water samples, test papers and DFT calculations. Thus, it was confirmed that **CFHZ** could be a promising probe for Fe^3+^ detection.

## Supplementary Information

Below is the link to the electronic supplementary material.

Supplementary file1 (DOCX 5765 KB)

## Data Availability

No datasets were generated or analysed during the current study.
