# Supplementary material for: A ″On–Off″ Fluorescent Sensor Based on Coumarin-Furoic Hydrazide for Recognition of Fe3+: Drinking Water, Test Strip Applications and DFT Calculations
Source: J Fluoresc. 2025 Feb 26;35(10):8937–48. doi: 10.1007/s10895-025-04212-2 (PMC12672624; doi:10.1007/s10895-025-04212-2)
Supplement: Supplementary file 1 — Supplementary file1 (DOCX 5765 KB) [file 10895_2025_4212_MOESM1_ESM.docx]

**Supporting Information**

**A ″on–off″ fluorescent sensor based on coumarin-furoic hydrazide for recognition of Fe^3+^: Drinking water, test strip applications and DFT calculations**

**Abdurrahman Karagoz^a,b^, Tahir Savran^a*^, Ibrahim Yilmaz^c,d*^**

*^a^ Department of Chemistry, Kamil Ozdag Science Faculty, Karamanoglu Mehmetbey University, 70100, Karaman, Türkiye*

*^b^ Department of Chemistry and Chemical Processing Technologies, Vocational School of Technical Sciences, Karamanoglu Mehmetbey University, 70100, Karaman, Türkiye*

*c Department of Mathematics and Science Education, Faculty of Education, Bolu Abant İzzet Baysal University, 14030, Bolu, Türkiye*

*d Innovative Food Technologies Development Application and Research Centre, Bolu Abant İzzet Baysal University, 14030, Bolu, Türkiye*

^*^Corresponding Author–1 : Dr. Tahir Savran (*Corresponding author–1*)

Address : ^a^Karamanoglu Mehmetbey University, Kamil Ozdag Science Faculty, Department of Chemistry, 70100, Karaman, Turkiye

Tel : +90 338 226 00 00 / 3861

e–mail : [tahir.savran@gmail.com](mailto:tahir.savran@gmail.com)

^*^Corresponding Author–2 : Prof. Dr. Ibrahim Yilmaz (*Corresponding author–2*)

Address : ^c^Bolu Abant İzzet Baysal University, Education Faculty, Department of Mathematics and Science Education, 14030, Bolu, Türkiye

Tel : +90 374 254 10 00 / 5697

e–mail : [iyilmaz33@ibu.edu.tr](mailto:iyilmaz33@ibu.edu.tr)

Author–3 : Dr. Abdurrahman Karagoz (*author–3*)

Address : ^a^Karamanoglu Mehmetbey University, Vocational School of Technical Sciences, Department of Chemistry and Chemical Processing Technologies, 70100, Karaman, Turkiye

Tel : +90 338 226 00 00 / 2215-4154

e–mail : [karagoz70@gmail.com](mailto:karagoz70@gmail.com)

**Captions**

**Fig S1.** ^1^H–NMR spectrum of the **CFHZ** in DMSO-*d_6_*

**Fig S2.** ^13^C–NMR spectrum of the **CFHZ** in DMSO-*d_6_*

**Fig S3.** MALDI TOF–MS spectrum of the **CFHZ**

**Fig S4.** MALDI TOF–MS spectrum of the **CFHZ**

**Fig S5.** FTIR spectra of the **CFHZ** and **CFHZ-Fe^3+^**

**Fig S6.** Emission spectra of **CFHZ**, **CFHZ**−Fe^3+^ (a) in different solvents and (b) Ethanol-Water binary mixture, in the presence and absence of Fe^3+^ at different ratios for **CFHZ**

**Fig S7.** pH study for **CFHZ** and **CFHZ** –Fe^3+^ in EtOH: H_2_O (99:1, v:v)

**Fig S8.** Response time graph of the chemoprobe **CFHZ** towards Fe^3+^

**Fig S9.** Reversibility study of **CFHZ** with the adding of EDTA (10^-2^ M) to **CFHZ–Fe^3+^**

**Fig S10.** Photographs taken under UV lamp for **CFHZ** test papers in EtOH: H_2_O (99:1, v:v), (a) with only **CFHZ** solution added (b) with **CFHZ** - Fe^3+^ solution added

**Synthesis of 3-metyl-2-oxo-2H-chromen-7-yl propionate 3 and 7-hydroxy-2-oxo-2H-chromene-3-carbaldehyde 4**

A mixture of 2,4-dihydroxybenzaldehyde 3.00 g, sodium propionate 4.50 g, propionic anhydride 7.54 g and piperidine 0.4 ml was stirred under reflux for 6 h. The reaction solution was poured into ice water, the precipitate was collected by filtration and dissolved in EtOAc, the organic layer was washed with water and 1 N HCl. Then it was concentrated and dried over anhydrous Na2SO4. The resulting residue was subjected to column chromatography and white crystals of compound **3** in 50 % yield were obtained. ^1^H NMR (400 MHz, CDCl_3_) δ7.50 (s, 1H), 7.40 (d, 1H, *J* = 8 Hz), 7.08 (d, 1H, *J* = 2 Hz), 7.00 (dd, 1H, *J* = 8, 2 Hz), 2.64-2.59 (2H), 2.20 (s, 3H), 1.29-1.25 (3H). 13C NMR (100 MHz, CDCl3) δ 172.5, 162.0, 153.8, 152.3, 138.9, 127.7, 125.2, 118.4, 117.4, 110.1, 27.8, 17.2, 9.1

To a solution of compound **3** (2.7 g, 11.9 mmol) in 75 mL of CCl_4_ was added NBS (5.31 g, 29 mmol) and a trace amount of AIBN, and the mixture was then refluxed. After reaction for 8 h, the solvent was removed under reduced pressure. To the resulting residue were added NaOAc (8.78 g, 107 mmol) and acetic acid (80 mL), and the mixture was then heated to reflux for 12 h. Subsequently, 2 N HCl (60 mL) was added to the hot reaction mixture, and the reaction was allowed to continue for 30 min and left to stir at room temperature overnight. The reaction mixture was then evaporated to dryness, and the residue was directly purified by column chromatography to afford **4** in 50 % yield as a yellow powder. 1H NMR (400 MHz, DMSO-*d*6) δ 10.86 (s, 1H), 8.20 (s, 1H), 7.89 (d, 1H, *J* = 8.2 Hz), 7.23 (dd, 1H, *J* = 8.2, 2 Hz), 7.21 (d, 1H, *J* = 2 Hz). 13C NMR (50 MHz, DMSO-*d*6) δ 188.2, 165.3, 160.1, 158.0, 147.7, 133.8, 117.6, 114.9, 111.3, 102.7.

**Fig S1.**  ^1^H–NMR spectrum of the **CFHZ** in DMSO-*d_6_*

**Fig S2.**  ^13^C–NMR spectrum of the **CFHZ** in DMSO-*d_6_*


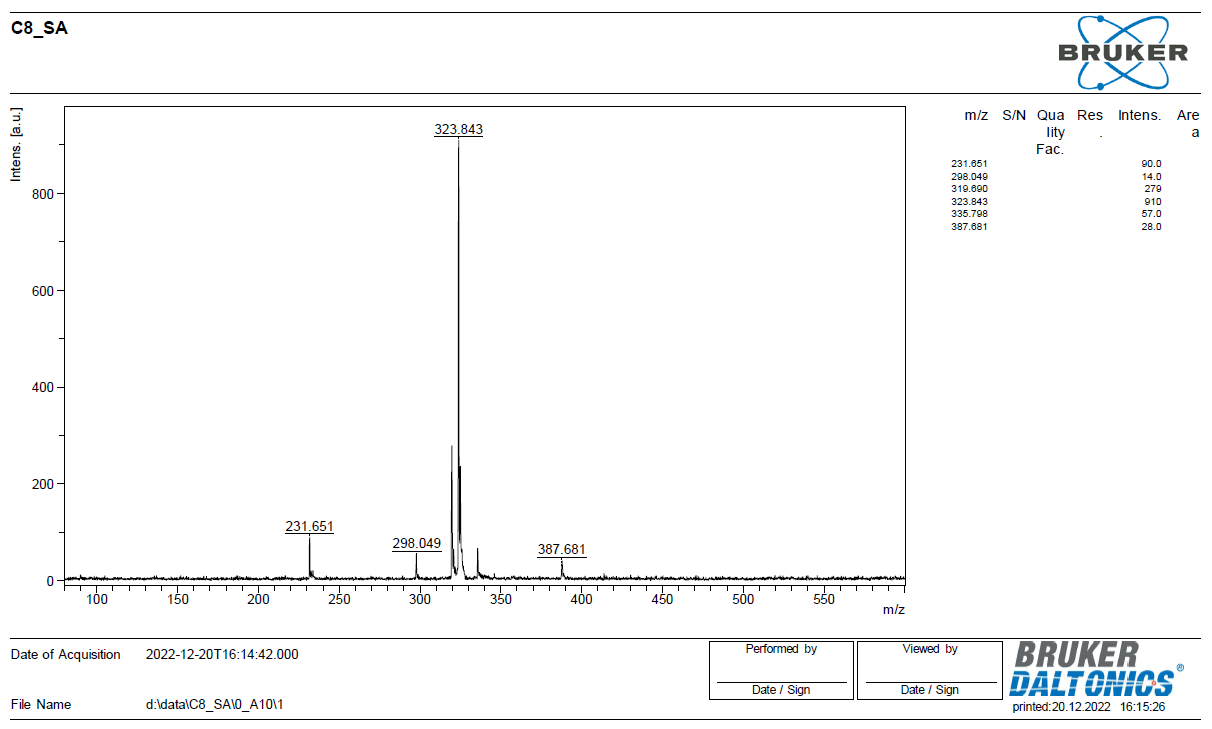


**Fig S3.**  MALDI TOF–MS spectrum of the **CFHZ**


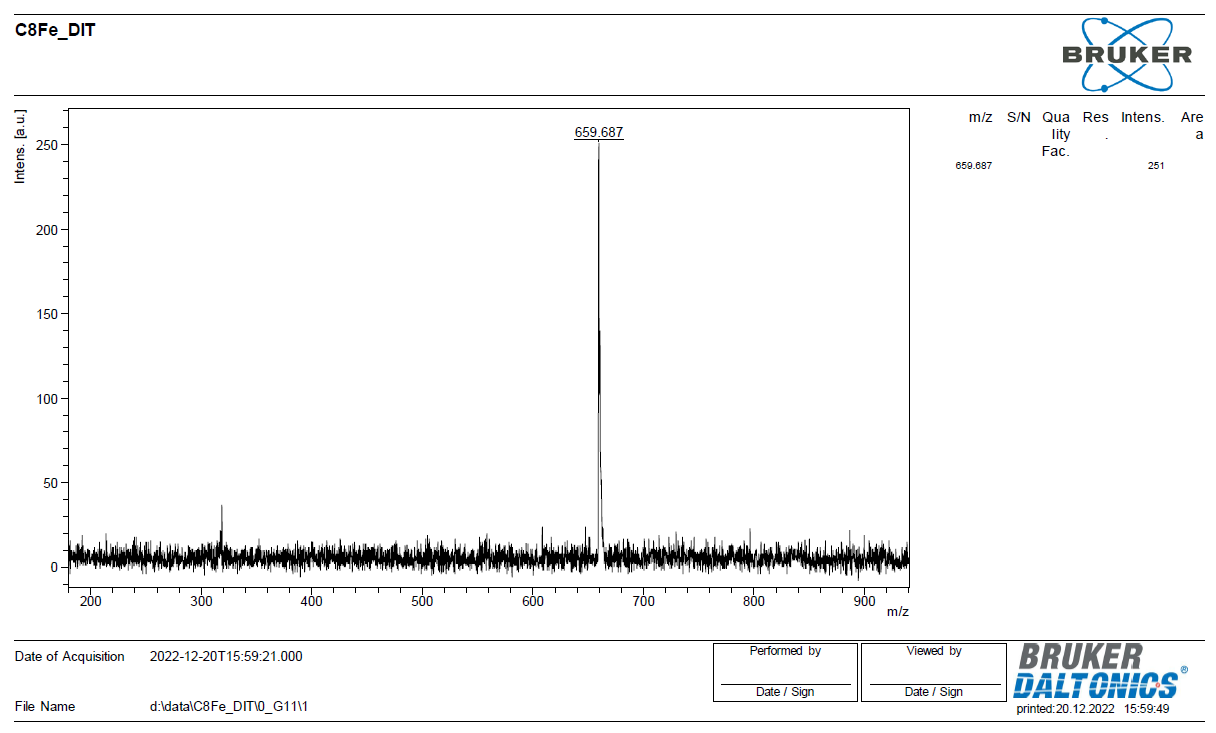


**Fig S4.**  MALDI TOF–MS spectrum of the **CFHZ-Fe^3+^**





**Fig S5.** FTIR spectra of the **CFHZ** and **CFHZ-Fe^3+^**


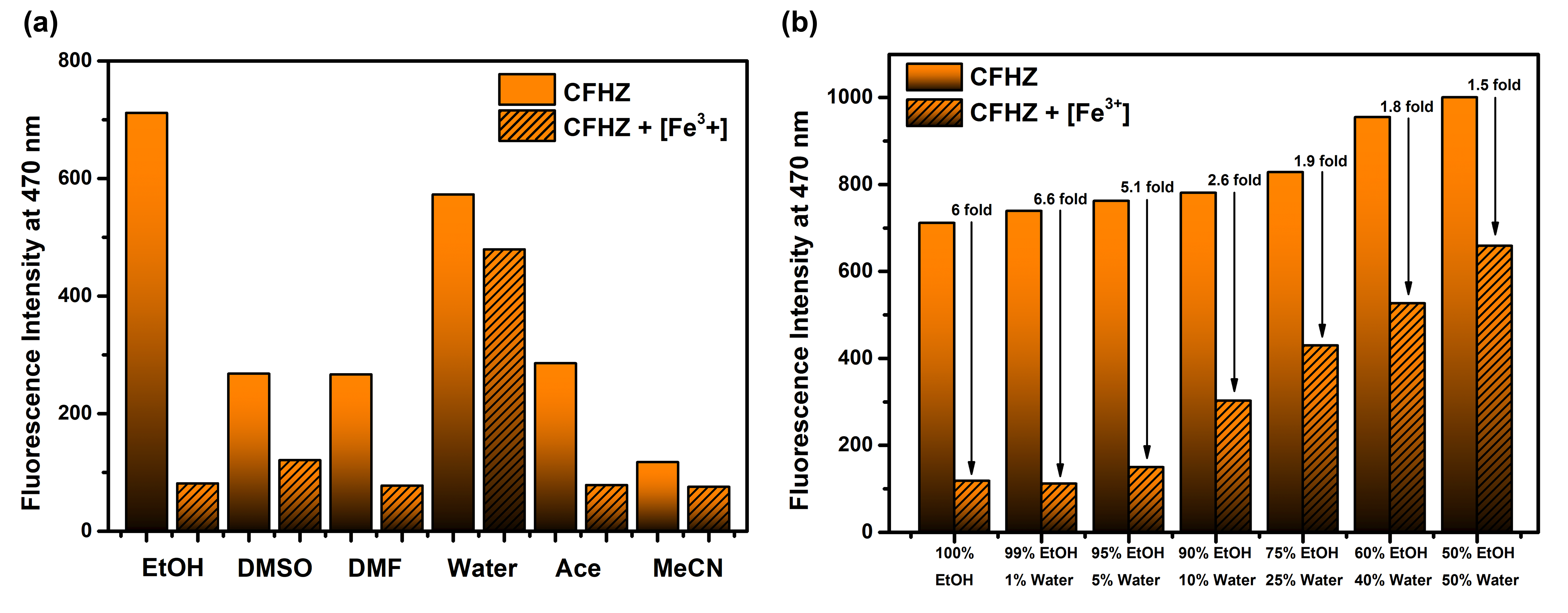


**Fig S6.** Emission spectra of **CFHZ**, **CFHZ**−Fe^3+^ (a) in different solvents and (b) Ethanol-Water binary mixture, in the presence and absence of Fe^3+^ at different ratios for **CFHZ**.





**Fig S7.** pH study for **CFHZ** and **CFHZ** –Fe^3+^ in EtOH: H_2_O (99:1, v:v)


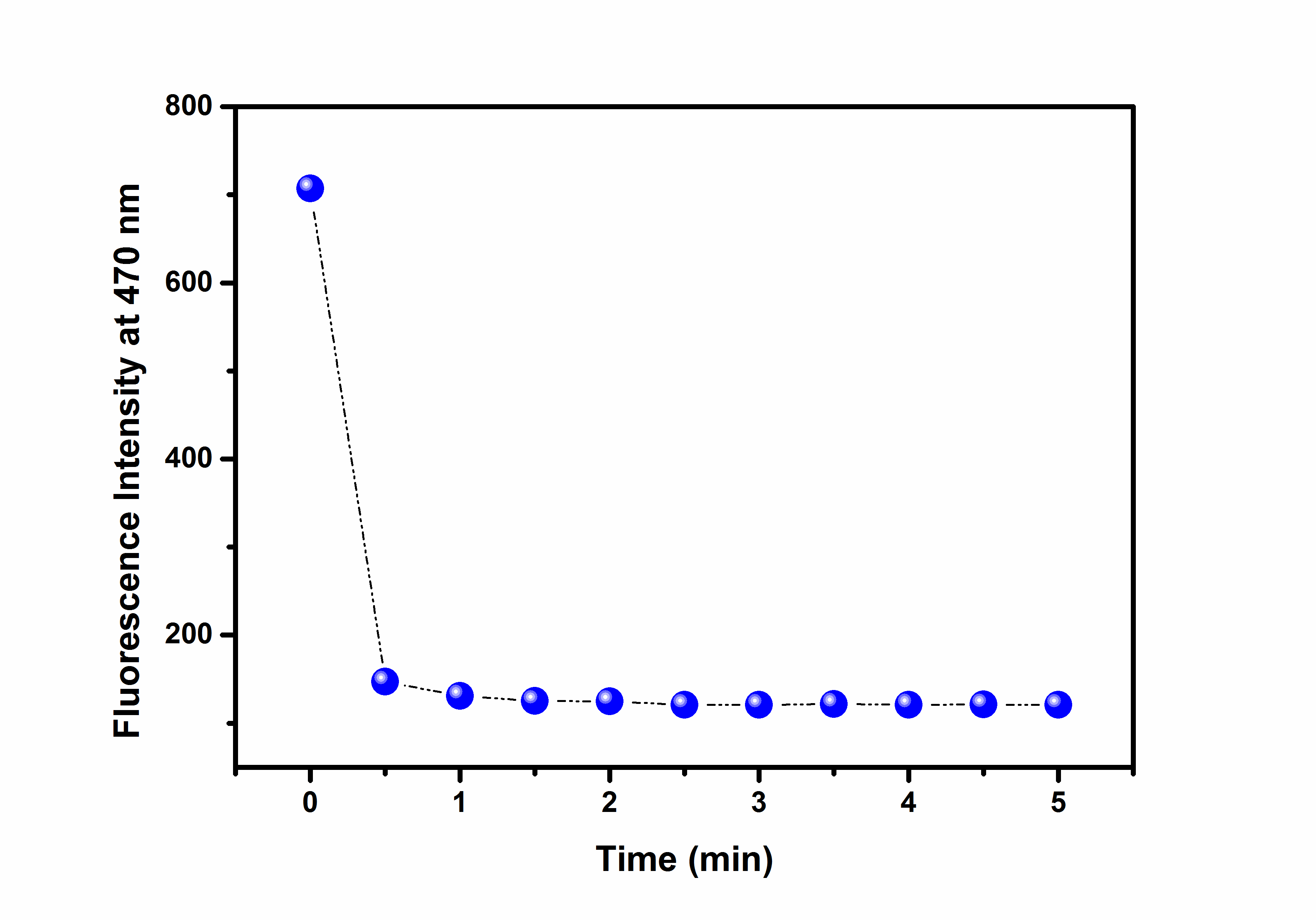


**Fig S8.** Response time graph of the chemoprobe **CFHZ** towards Fe^3+^


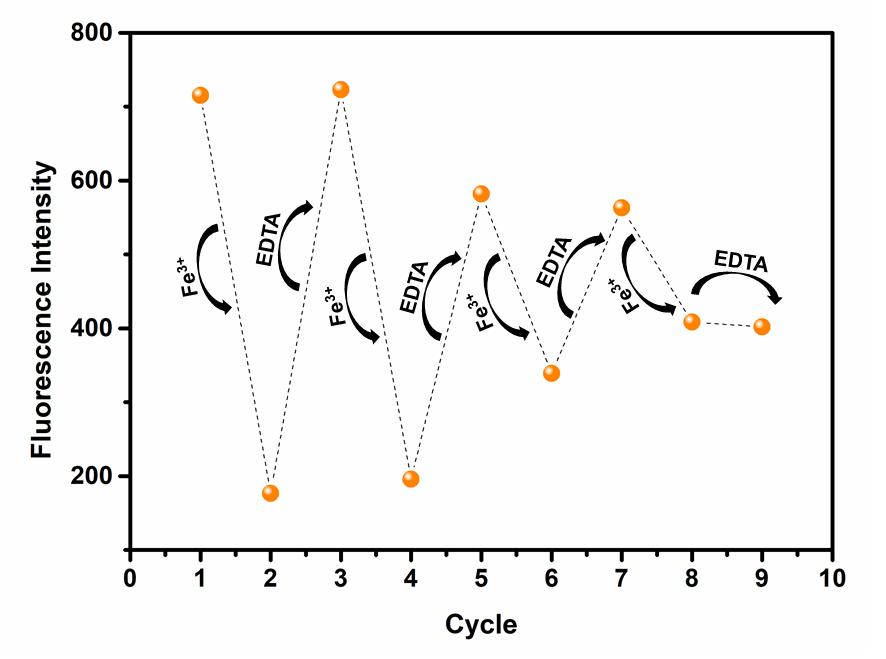


**Fig S9.** Reversibility study of **CFHZ** with the adding of EDTA (10^-2^ M) to **CFHZ–Fe^3+^**


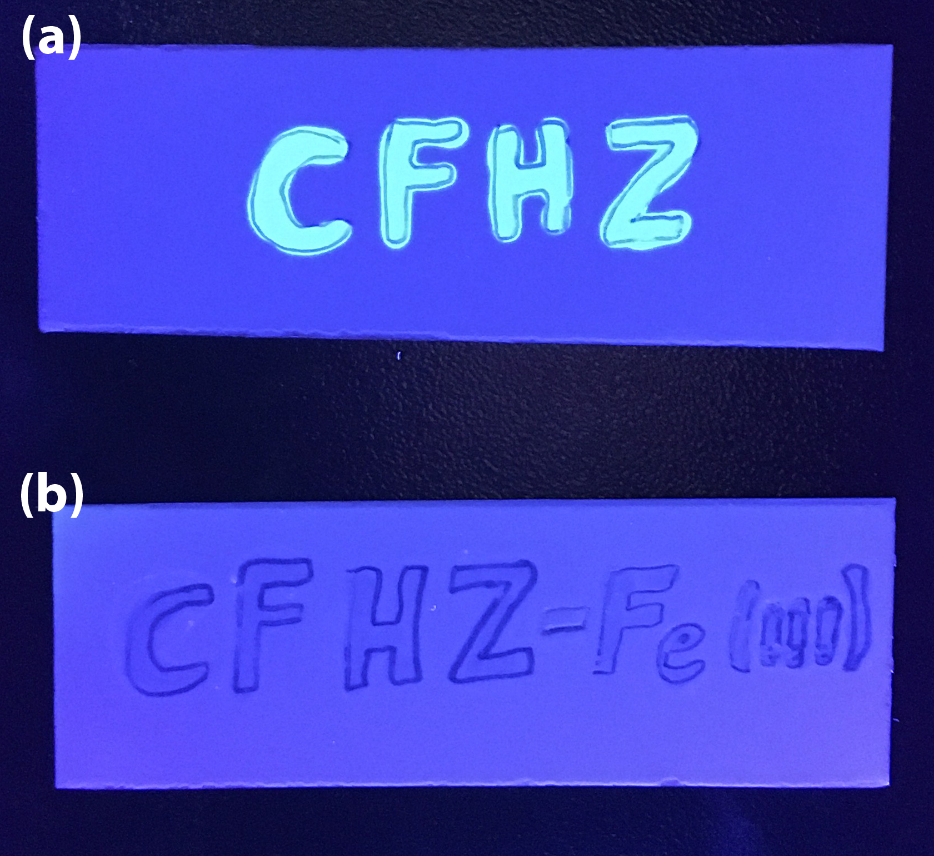


**Fig S10.** Photographs taken under UV lamp for **CFHZ** test papers in EtOH: H_2_O (99:1, v:v), (a) with only **CFHZ** solution added (b) with **CFHZ** - Fe^3+^ solution added
